# Supplementary material for: Association between oral microbiome and seven types of cancers in East Asian population: a two-sample Mendelian randomization analysis
Source: Front Mol Biosci. 2023 Nov 21;10:1327893. doi: 10.3389/fmolb.2023.1327893 (PMC10702768; doi:10.3389/fmolb.2023.1327893)
Supplement: Supplementary file 1 [file DataSheet1.ZIP › Supplementary Materials/MR plots of saliva/saliva═╝/Lung Cancer/pheno.2655_to_lung cancer_leave_one_out.pdf]

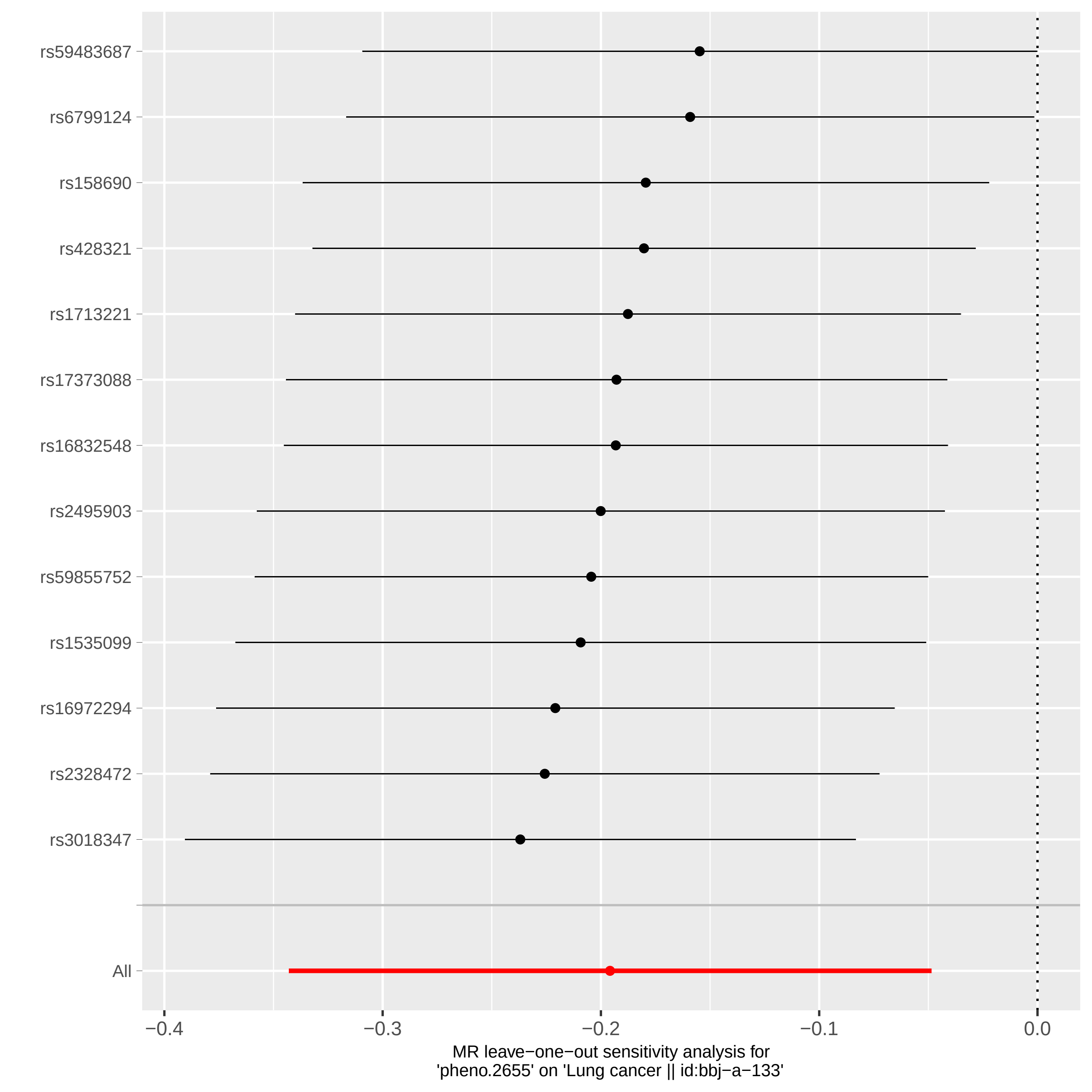

rs59483687
rs6799124
rs158690
rs428321
rs1713221
rs17373088
rs16832548
rs2495903
rs59855752
rs1535099
rs16972294
rs2328472
rs3018347
All
−0.4
−0.3
−0.2
−0.1
0.0
MR leave−one−out sensitivity analysis for
'pheno.2655' on 'Lung cancer || id:bbj−a−133'
